# Protocol for a prospective magnetic resonance imaging study on supraspinal lower urinary tract control in healthy subjects and spinal cord injury patients undergoing intradetrusor onabotulinumtoxinA injections for treating neurogenic detrusor overactivity

**DOI:** 10.1186/1471-2490-14-68

**Published:** 2014-08-18

**Authors:** Lorenz Leitner, Matthias Walter, Patrick Freund, Ulrich Mehnert, Lars Michels, Spyros Kollias, Thomas M Kessler

**Affiliations:** 1 Neuro-Urology, Spinal Cord Injury Centre & Research, University of Zürich, Balgrist University Hospital, Forchstrasse 340, 8008 Zürich, Switzerland; 2 Institute of Neuro-Radiology, University of Zürich, University Hospital Zürich, Zürich, Switzerland

**Keywords:** Urinary bladder, Spinal cord injury, Neuroimaging, Magnetic resonance imaging, Neurogenic detrusor overactivity, OnabotulinumtoxinA intradetrusor injections

## Abstract

**Background:**

The control of the lower urinary tract is a complex, multilevel process involving both the peripheral and central nervous system. Due to lesions of the neuraxis, most spinal cord injury patients suffer from neurogenic lower urinary tract dysfunction, which may jeopardise upper urinary tract function and has a negative impact on health-related quality of life. However, the alterations to the nervous system following spinal cord injury causing neurogenic lower urinary tract dysfunction and potential effects of treatments such as intradetrusor onabotulinumtoxinA injections on lower urinary tract control are poorly understood.

**Methods/Design:**

This is a prospective structural and functional magnetic resonance imaging study investigating the supraspinal lower urinary tract control in healthy subjects and spinal cord injury patients undergoing intradetrusor onabotulinumtoxinA injections for treating neurogenic detrusor overactivity.

Neuroimaging data will include structural magnetic resonance imaging (T1-weighted imaging and diffusion tensor imaging) as well as functional, i.e. blood oxygen level-dependent sensitive magnetic resonance imaging using a 3 T magnetic resonance scanner. The functional magnetic resonance imaging will be performed simultaneously to three different bladder stimulation paradigms using an automated magnetic resonance compatible and synchronised pump system.

All subjects will undergo two consecutive and identical magnetic resonance imaging measurements. Healthy subjects will not undergo any intervention between measurements but spinal cord injury patients will receive intradetrusor onabotulinumtoxinA injections for treating neurogenic detrusor overactivity.

Parameters of the clinical assessment including bladder diary, urinalysis, medical history, neuro-urological examination, urodynamic investigation as well as standardised questionnaires regarding lower urinary tract function and quality of life will serve as co-variates in the magnetic resonance imaging analysis.

**Discussion:**

This study will identify structural and functional alterations in supraspinal networks of lower urinary tract control in spinal cord injury patients with neurogenic detrusor overactivity compared to healthy controls. Post-treatment magnetic resonance imaging measurements in spinal cord injury patients will provide further insights into the mechanism of action of treatments such as intradetrusor onabotulinumtoxinA injections and the effect on supraspinal lower urinary tract control.

**Trial registration:**

ClinicalTrials.gov NCT01768910.

## Background

Spinal cord injury (SCI) is a devastating event with far-reaching consequences for the individual’s health and the economic and social future. In the past, renal failure due to neurogenic lower urinary tract dysfunction (NLUTD) was a leading cause of death after SCI [1]. Furthermore, NLUTD has a highly negative impact on patients’ quality of life (QoL). Acute SCI initially causes “spinal shock”, characterised by an acontractile/hypocontractile detrusor and urinary retention, which in case of a suprasacral lesion (today the vast majority of SCI) is followed by development of detrusor overactivity mostly combined with detrusor sphincter dyssynergia [2]. Antimuscarinics are the pharmacological first-line treatment for detrusor overactivity although the effectiveness is limited [3]. In addition, many patients discontinue antimuscarinics due to bothersome side-effects [4]. Thus, intradetrusor onabotulinumtoxinA injections have become an established, highly effective, minimally invasive, and generally well-tolerated therapy for refractory detrusor overactivity [5] to improve patients’ health and QoL [6]. Despite the popularity of intradetrusor onabotulinumtoxinA injections, the exact mechanisms of action remain to be elucidated. Nevertheless, it seems highly probable that, in addition to a direct efferent effect by blocking the presynaptic release of acetylcholine from the parasympathetic innervation resulting in temporary chemodenervation of the detrusor, onabotulinumtoxinA also modulates afferent pathways [7]. It is, however, not known whether this treatment can normalise alterations in supraspinal areas and whether supraspinal modulation correlates with clinical improvements.

The control of the lower urinary tract (LUT) is a complex, multilevel process that involves both the peripheral and central nervous system but the exact mechanisms involved in humans are still incompletely understood [8]. Neuroimaging studies over the last decade have consistently pointed to a complex supraspinal network that controls LUT function [9]. These studies tremendously increased our understanding of how human LUT function is coordinated and how it can be affected by neurological disorders. Recent neuroimaging studies demonstrated reorganisation of supraspinal activity in response to LUT stimulation tasks in patients with disorders such as Parkinson’s disease [10-12] and SCI [13] as compared to healthy controls, which might represent the neural correlate of their NLUTD.

Cortical and sub-cortical (for example, brainstem) brain regions are essential for voluntary LUT control [9,14,15]. Investigation of the supraspinal regions with high-resolution imaging techniques, for example, structural magnetic resonance imaging (MRI) and functional MRI (fMRI), can significantly increase our knowledge on the effects of supraspinal lesions and alterations related to NLUTD [9,16]. Although diffusion tensor imaging (DTI) [17] is popular in other fields in neuroscience, it has only been applied in the context of supraspinal LUT control in one prospective study [18] in patients with non-neurogenic LUT symptoms.

In this study, we will first identify supraspinal areas associated with LUT control in SCI patients with neurogenic detrusor overactivity and healthy controls. Subsequently, we will investigate the effects of intradetrusor onabotulinumtoxinA injections on supraspinal areas. Task-related blood oxygen level-dependent (BOLD) fMRI will be used along structural MRI (T1-weighted and DTI). Moreover, we will examine volumetric parameters (for example, grey and white matter concentration) by voxel- (VBM) [19] and tensor-based morphometry (TBM) [20], structural integrity and connectivity of white matter tracts (DTI) as well as structural (SC) and functional connectivity (FC).

This unique and detailed multimodal imaging and clinical approach will distinguish the different structural and functional processing units involved during supraspinal LUT control and will identify dysfunctional neuronal components in SCI patients responsible for neurogenic detrusor overactivity.

For a test-retest validation, we will additionally investigate the reliability [21] of BOLD signals in task-related fMRI in healthy controls by the intra-class correlation coefficient (ICC) for absolute or consistent agreement of participants activations over two visits.

## Methods/Design

### Study design

This prospective research study will be conducted at the University of Zürich, Zürich, Switzerland in cooperation with our partner, the institute of Neuro-Radiology, University of Zürich, University Hospital Zürich, Zürich, Switzerland.

### Study location

The study has two study locations, that is, the department of Neuro-Urology, Spinal Cord Injury Centre & Research, University of Zürich, Balgrist University Hospital, Zürich, Switzerland (first and third visit, see below) and the MR-Centre, University Hospital Zürich, Zürich, Switzerland (second and third visit, see below).

### Study population and recruitment

In line with the inclusion and exclusion criteria (Table 1), we will investigate SCI patients with neurogenic detrusor overactivity and healthy controls with an unimpaired LUT function. Participants of both groups will be similar according to age and gender.

**Table 1:** Inclusion and exclusion criteria for all participants

**Groups**	**Inclusion criteria**	**Exclusion criteria**
All participants	• MR suitability	• Pregnancy or breast feeding
	• Written informed consent	• Any anatomical anomaly of LUT/genitalia
		• Any LUT malignancy
		• Claustrophobia
SCI patients	• Age limit: > 18 years	• Symptomatic UTI
	• Neurogenic detrusor overactivity	
	• Refractory to antimuscarinic treatment	
	• Scheduled for intradetrusor onabotulinumtoxinA injections	
Healthy controls	• Age limits: > 18 years	• Impaired LUT function
	• Unimpaired LUT function	• Any LUTS (3-day bladder diary)
	• No LUTS (3-day bladder diary)	• Any number of episodes of urinary urgency/week
	• No episode of urinary urgency/week	• Urinary frequency > 8/24 h
	• Urinary frequency < 8/24 h	• Any craniocerebral injury or surgery
		• Any permanent ferromagnetic implant
		• Any previous surgery of LUT/genitalia
		• UTI
		• PVR > 150 mL

LUT = lower urinary tract, LUTS = lower urinary tract symptoms, MR = magnetic resonance, PVR = post void residual, SCI = spinal cord injury, UTI = urinary tract infection.

20–24 SCI patients with neurogenic detrusor overactivity refractory to antimuscarinics and scheduled for study independent intradetrusor onabotulinumtoxinA injections and 12–24 healthy controls will be recruited.

### Interventions

**Figure 1:**
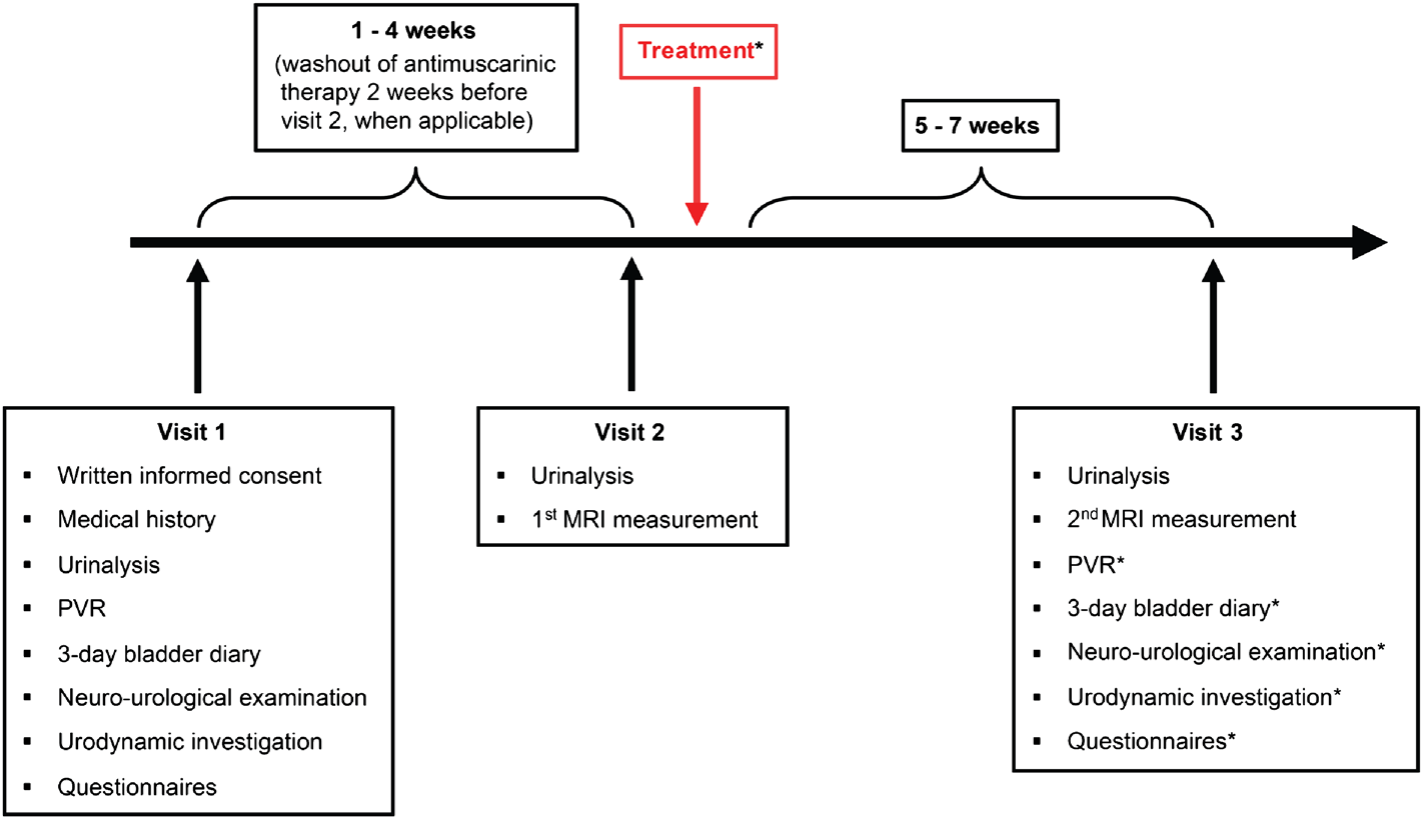
**Timetable and characteristics of all visits.** *Patients only, Treatment = intradetrusor onabotulinumtoxinA injection, MRI = magnetic resonance imaging, PVR = post void residual

**Figure 2:**
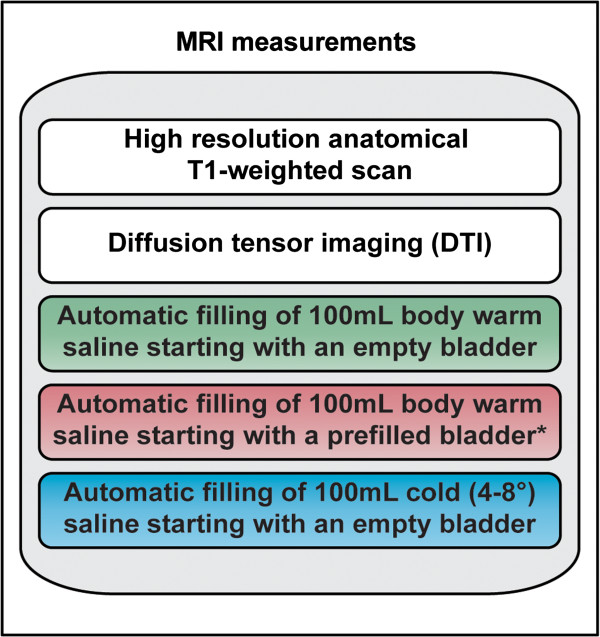
**Sequences of magnetic resonance imaging (MRI) measurements.** *Bladder will be filled with body warm saline until a persistent desire to void is present.

**Figure 3:**
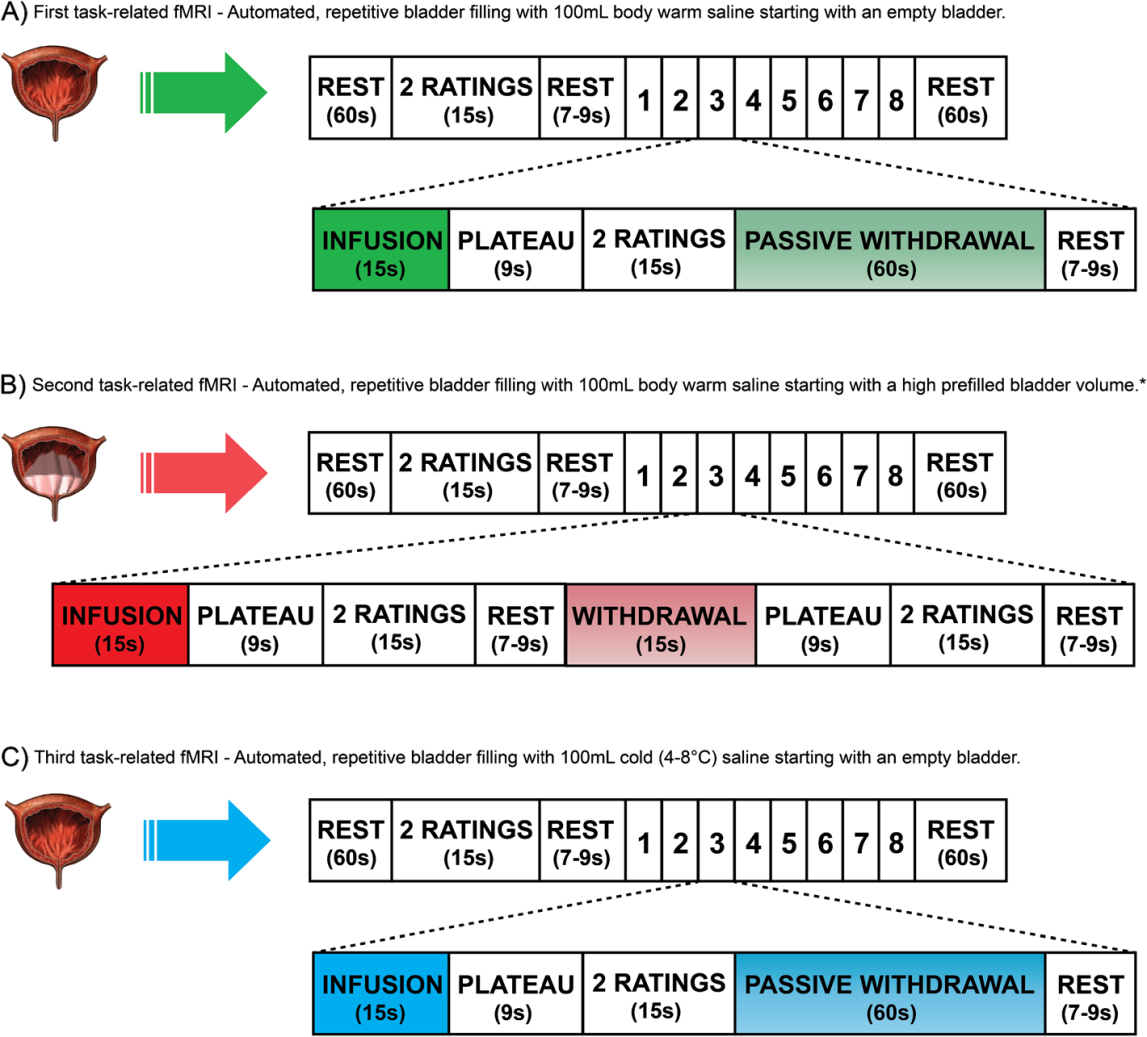
**Scan paradigm of three different task-related functional MRIs (fMRIs).** All task-related fMRIs identically start with a ‘baseline’ rest (60 s, no specific stimulus or task is performed), a ‘baseline’ rating (desire to void and level of pain), a short rest (jittered between 7 and 9 s in which blood oxygen level-dependent (BOLD) activation resulting from motor activity during the previous rating will return to baseline to avoid contamination of the following condition) and conclude with a ‘last’ rest (60 s, no specific stimulus or task is performed). All task-related fMRIs consist of eight repetitive blocks, each with either five (first and third fMRIs) or eight (second fMRI) conditions. **(A)** Conditions of the first task-related fMRIs: (1) automated infusion of 100 mL body warm saline, (2) plateau phase (bladder distention after infusion is perceived), (3) rating, (4) passive withdrawal to empty the bladder completely and (5) short rest. This task-related fMRI starts with an empty bladder. **(B)** Conditions of the second task-related fMRIs: (1) automated infusion of 100 mL warm saline, (2) plateau phase, (3) rating, (4) short rest, (5) automated withdrawal of 100 mL, (6) plateau phase (bladder distention after withdrawal is perceived), (7) rating and (8) short rest. *This task-related fMRI (B) starts with a high prefilled bladder volume, that is, the bladder will be filled with body warm saline until a persistent desire to void is present. **(C)** Conditions of the third task-related fMRIs: (1) automated infusion of 100 mL cold (4–8°C) saline, (2) plateau phase, (3) rating, (4) passive withdrawal to empty the bladder completely and (5) short rest. This task-related fMRI starts with an empty bladder.

Subjects providing written informed consent will be invited for the following visits (Figure 1):

Screening (Visit 1): Evaluation for study eligibility will be based on medical history, urinalysis to exclude urinary tract infection (UTI) and pregnancy in female participants, 3-day bladder diary, post void residual, urodynamic parameters as well as on standardised validated questionnaires such as Qualiveen [22] and International Consultation on Incontinence modular questionnaire [(ICIQ), Bristol Urological Institute, Southmead Hospital Bristol, UK] assessing lower urinary tract symptoms (LUTS) in both woman (ICIQ-FLUTS) and men (ICIQ-MLUTS).MRI measurements (Visit 2 and 3, Figure 1): Two MRI measurements will be performed in identically manner within a 5 to 7 weeks interval.Both MRI measurements will be performed using a Philips Ingenia 3 Tesla MR scanner (Philips Medical Systems, Best, The Netherlands) with a 16-channel head coil to acquire the following sequences (Figure 2):

• Structural sequences will comprise T1-weighted and DTI.• Functional sequences will comprise three different task-related fMRI paradigms (Figure 3).

• In the first two task-related fMRI paradigms, we will examine the effect of visceral bladder sensation by automated, repetitive bladder filling with 100 mL body warm (37°C) saline starting with an empty bladder (first paradigm) and a bladder volume eliciting desire to void (second paradigm).

• The third task-related fMRI paradigm will contain automated, repetitive filling of 100 mL cold (4-8°C) saline starting with an empty bladder.

Repetitive filling will be performed using an automated MR-compatible and MR-synchronised pump system [18] to precisely fill and drain the bladder. During the MRI measurements, all participants will use an MR-compatible handheld response system [23] to rate their desire to void and level of pain on a displayed visual analogue scale.

### Study outcome measures

#### Primary

(A) Task-related BOLD signal intensity in supraspinal regions of interest (ROI) in SCI patients with neurogenic detrusor overactivity compared to healthy controls.

(B) Supraspinal morphometry of ROIs in SCI patients with neurogenic detrusor overactivity compared to healthy controls.

(C) SC and FC between ROIs of the supraspinal LUT controlling circuitries in SCI patients with neurogenic detrusor overactivity and healthy controls.

(D) Changes of task-related BOLD signal intensity, SC and FC between ROIs in SCI patients with neurogenic detrusor overactivity before and after study independent intradetrusor onabotulinumtoxinA injections.

#### Secondary

(A) Reliability of BOLD signal changes between first and second MRI measurement in healthy controls.

(B) Correlations between clinical co-variates that are bladder diary parameters, urodynamic parameters, level of desire to void during fMRI and task-related BOLD signal intensity in supraspinal ROIs as well as structural parameters, that is, grey matter volume and number of white matter tracts between ROIs.

### Data analysis

Clinical data, for example, questionnaires scores, urodynamic parameters and 3-day bladder diary outcomes will be statistically analysed and compared between groups using IBM’s Statistical Package for the Social Sciences (SPSS) version 19.0 or newer (Armonk, New York, U.S.). The statistical analysis will be presented with means and standard deviations or with medians and interquartile ranges as appropriate.

For the analysis of the neuroimaging data, we will use statistical parametric mapping (SPM) V.8 or newer (Wellcome Department of Imaging Neuroscience, University College London, UK) and toolboxes as appropriate.

### Regulatory issues

#### Ethical approval

This study has been approved by the local ethics committee (Kantonale Ethikkommission Zürich, KEK-ZH-Nr. 2011–0346) and will be performed in accordance to the World Medical Association Declaration of Helsinki [24], the guidelines for Good Clinical Practice (GCP) [25], and the guidelines of the Swiss Academy of Medical Sciences [26]. Handling of all personal data will strictly comply with the federal law of data protection in Switzerland [27].

#### Safety

According to the safety regulations of the MR-Centre of the University Hospital Zürich, the staff involved in this study will be instructed and trained. Prior to entering the scanner room, all participants will be asked to remove any ferromagnetic items (for example, bra, chains, earrings, rings, and piercings). All participants will be provided with standardised clinical scrubs to prevent incidental import of ferromagnetic items into the MR room. To exclude UTI or pregnancy, urinalysis will be performed on every participant before urodynamic investigations and MR measurements. In case of pregnancy, the participant will be excluded from the study and referred to a gynaecologist. In case of UTI, the participant will not undergo the experiment but will be treated appropriately. After successful treatment, a reassignment to the study is possible.

In case of an adverse event (AE) or a severe adverse event (SAE), as defined by the International Organization for Standardization (ISO, 14155) [28] and the International Conference on Harmonisation (ICH) GCP guidelines (E6) [25], responsible authorities, that is, the principle investigator and the ethics committee will be informed. Appropriate actions will be executed. All AEs and SAEs will be followed as long as medically indicated.

#### Funding

The Swiss National Science Foundation (grant number: 135774), Wings for Life, the Emily Dorothy Lagemann Stiftung and the Swiss Continence Foundation are funding this study.

## Discussion

This study will investigate structural and functional abnormalities and specific alterations in the brain networks of supraspinal LUT control in SCI patients with neurogenic detrusor overactivity compared to healthy controls using a multimodal imaging protocol. Importantly, effects on the supraspinal LUT control after treatment for neurogenic detrusor overactivity with intradetrusor onabotulinumtoxinA injections in SCI patients will be explored.

The findings will help to verify, amend, or adjust neuronal circuitry models established from findings in healthy controls, now in the context of SCI patients with neurogenic detrusor overactivity. Furthermore it will show whether neurogenic detrusor overactivity specific treatment such as intradetrusor onabotulinumtoxinA injections induce structural or functional reorganisation in supraspinal areas related to LUT control and in how far such changes correlate with improvements of clinical outcome parameters. These investigations will help us to understand how onabotulinumtoxinA modulates afferent pathways.

Advanced neuroimaging and evaluation techniques have the potential to serve as quantifiable outcome measures for therapy success and for improving our treatment strategies in this patient population.

### Trial status

The trial is in the recruiting phase at the time of manuscript submission.

## Abbreviations

AE: Adverse event; BOLD: Blood oxygen level-dependent; DTI: Diffusion tensor imaging; FA: Fractional anisotropy; FC: Functional connectivity; fMRI: Functional magnetic resonance imaging; GLM: General linear model; GCP: Good clinical practice; ICC: Intra-class correlation coefficient; ICH: International conference on harmonisation; ISO: International organization for standardization; LUT: Lower urinary tract; LUTS: Lower urinary tract symptoms; MR: Magnetic resonance; MRI: Magnetic resonance imaging; MD: Mean diffusivity; MNI: Montreal neurologic institute; NLUTD: Neurogenic lower urinary tract dysfunction; PVR: Post void residual; QoL: Quality of life; ROI: Regions of interest; SAE: Severe adverse event; SPM: Statistical parametric mapping; SC: Structural connectivity; SCI: Spinal cord injury; TBM: Tensor-based morphometry; UTI: Urinary tract infection; VBM: Voxel-based morphometry.

## Competing interests

The authors declare that they have no competing interests.

## Authors’ contributions

All authors participated in creating the study design. LL, MW, and TMK drafted the manuscript. PF, UM, LM, and SK provided a critical revision of the manuscript. PF, UM, SK and TMK obtained the funding of this study. All the authors read and approved the final manuscript.

## Pre-publication history

The pre-publication history for this paper can be accessed here:

http://www.biomedcentral.com/1471-2490/14/68/prepub
